# Hypoxia-Inducible Factor-1*α* Expression in Indonesian Laryngeal Squamous Cell Carcinoma Patients

**DOI:** 10.1155/2016/3215463

**Published:** 2016-11-01

**Authors:** Agus Surono, Priyanto Priyanto, Sagung Rai Indrasari

**Affiliations:** Department of Otorhinolaryngology Head and Neck Surgery, Faculty of Medicine, Universitas Gadjah Mada, Yogyakarta, Indonesia

## Abstract

*Objectives*. This research aimed to determine the association between hypoxia-inducible factor-1*α* (HIF-1*α*) expression and laryngeal squamous cell carcinoma clinical stage.* Methods*. We retrospectively analyzed paraffin-embedded tissue from 47 laryngeal squamous cell carcinoma (LSCC) patients from 2011 to 2014. HIF-1*α* expression was analyzed by immunohistochemistry using an anti-HIF-1*α* mouse monoclonal antibody. The association between HIF-1*α* expression and clinical stage was analyzed using the chi square test.* Results*. The glottis was the predominant site of laryngeal squamous cell carcinoma occurrence, and 43/47 (91.5%) patients presented at an advanced stage. Of the advanced stage patients, 27/43 stained positive for HIF-1*α* expression and 16/43 stained negative. Of the early stage patients, 2/4 stained positive for HIF-1*α* expression and 2/4 stained negative. Statistical analysis did not demonstrate significant association of HIF-1*α* expression.* Conclusion*. There was no statistically significant association between HIF-1*α* expression and the clinical stage or histological differentiation of LSCC.

## 1. Introduction

Laryngeal cancer is common worldwide; it is the third most common head and neck malignancy, after nasopharyngeal and sinonasal tumors [1]. Every year, approximately 12,760 new cases of laryngeal cancer are diagnosed in the United States, and estimated 3560 deaths are caused by the disease [2]. The most common type of laryngeal cancer is squamous cell carcinoma (SCC), which can range from carcinoma* in situ* to poorly differentiated carcinoma. Laryngeal cancer is three times more likely to arise in the glottis than the supraglottis; cancer in the subglottis is extremely rare and accounts for just 2% of all laryngeal cancers [3].

A number of factors are believed to contribute to survival after laryngeal cancer diagnosis; the tumor stage, tumor site, treatment strategy, and patient's age and comorbidities are all thought to play a role [4]. Tumor hypoxia is a characteristic of many solid tumors. The causes of hypoxia are multifactorial and include abnormal or chaotic tumor vasculature, impaired blood perfusion, reduced oxygen consumption, and anemia [5]. Severe tumor hypoxia ultimately leads to tissue necrosis, but nonlethal levels of hypoxia can impact tumor cell biology. Hypoxia-inducible factor-1*α* (HIF-1*α*) is a transcription factor that mediates adaptive responses to hypoxia. HIF-1*α* activity is increased as a result of genetic alteration or intratumoral hypoxia in many human cancers. HIF-1*α* activates gene transcription to increase oxygen availability; HIF-1*α* can stimulate angiogenesis or reprogram cellular metabolism to adapt to reduced oxygen availability [6]. The regulation of HIF-1*α* subunits forms part of the oxygen response pathway regulation. In the presence of oxygen, the HIF-1*α* subunits are hydroxylated and are consequently degraded. However, in hypoxic conditions, they are not hydroxylated; HIF-1*α* is stabilized and can stimulate gene expression. HIF-1*α* regulates several important biological pathways, including those involved in cellular proliferation, angiogenesis, cell metabolism, apoptosis, and migration [7]. However, the role of HIF-1*α* activity in laryngeal cancer is poorly understood, and very few studies regarding HIF-1*α* in Indonesian laryngeal cancer patients have been published. The aim of this study was to determine HIF-1*α* expression in laryngeal SCC (LSCC).

## 2. Material and Method

The Ethics Committee of Faculty of Medicine of Universitas Gadjah Mada, Yogyakarta, approved this cross-sectional study. The study included paraffin-embedded tissue from 47 histologically diagnosed LSCC patients that were seen from January 2010 to April 2014. The study was conducted by the Departments of Otorhinolaryngology Head and Neck Surgery and Anatomical Pathology from the Faculty of Medicine at Universitas Gadjah Mada, Yogyakarta, Indonesia. The inclusion criteria were a patient age > 40 years and no previous chemotherapy, radiotherapy, or surgery. Patients with incomplete data or severe complications were excluded from the study.

Sections of 4-5 *μ*m were cut from the paraffin-embedded tissue blocks. The sections were deparaffinized in xylene and rehydrated through graded ethanol. Antigen retrieval was performed in a microwave oven with two cycles of 10 minutes. Endogenous peroxidase activity was blocked by incubating the slides in 1.5% hydrogen peroxide in absolute methanol at room temperature for 10 minutes. A mouse monoclonal immunoglobulin G (IgG) anti-HIF-1*α* antibody (R&D Systems, USA) was used to detect HIF-1*α* protein expression in the nucleus and cytoplasm. Primary antibodies were applied for 1 hour at room temperature and sections were washed three times with 50 mM Tris-buffered saline, pH 7.2 (TBS), prior to incubation with 50 *μ*L of a polymerized, horseradish peroxidase conjugated, anti-mouse IgG antibody for 30 minutes at room temperature. Sections were washed three times with 50 mM TBS and protein expression was visualized using diaminobenzidine (DAB Kit, Thermo Scientific, USA). Sections were subsequently counterstained with hematoxylin and eosin, dehydrated, and evaluated using a light microscope. Samples were incubated with 10 mM TBS instead of a primary antibody as a negative control. HIF-1*α* expression in the nucleus and cytoplasm was only scored as positive (1+) or negative (0). Positive staining was defined as being HIF-1*α* expression in >10% of the tumor area. The associations between HIF-1*α* expression and clinical stage and differentiation of LSCC were analyzed using the chi square test.

## 3. Results

Included in the study were 47 histologically diagnosed LSCC patients. The clinical stage of the patients was determined by computed tomography scans, chest X-rays, and abdominal ultrasound imaging. The patient characteristics, including their gender, age, clinical stage, and histopathological differentiation (well, moderate, or poor) are shown in Table 1. There were 24 (51.1%) patients that were <60 years old and 23 (48.9%) patients that were ≥60 years old. Positive HIF-1*α* staining and negative HIF-1*α* staining were observed in 29/47 (61.7%) and 18/48 (38.3%) of patients, respectively. Of the 29 HIF-1*α*-positive samples, 28/29 (96.5%) of the patients were male and 1 (3.5%) patient was female. The patients' clinical stage was defined as early (stages I and II) or advanced (stages III and IV). There were 4 (8.5%) early stage patients and 43 (91.5%) advanced stage patients. The primary tumor location, defined as being either the glottis or supraglottis, was predominantly the glottis; there were 42 (89.4%) patients with cancer in the glottis. Histopathological analysis indicated that 15/47 (31.9%) tumors were well differentiated, 24/47 (51.1%) tumors were moderately differentiated, and 8/47 (17.0%) tumors were poorly differentiated. No statistically significant difference in HIF-1*α* expression was observed in tumors of different differentiations (Table 2).

We proceeded to examine the association between HIF-1*α* expression and LSCC clinical stage. Of the 4 (8.5%) early stage patients, 2 patients were positive for HIF-1*α* protein expression and 2 were negative for HIF-1*α* expression. In the 43 (91.5%) advanced stage patients, there were 27 (62.8%) patients that were positive for HIF-1*α* protein expression and 16 (37.2%) patients that were negative for HIF-1*α* expression. However, the statistical analysis did not indicate any significant associations between HIF-1*α* expression and LSCC clinical stage (*p* = 0.631; Table 2).

## 4. Discussion

Previous studies have reported inconsistent results regarding the association between HIF-1*α* expression and LSCC clinical stage. Wu et al. [8] found a significant relationship between HIF-1*α* expression and a T3-T4 stage LSCC (*p* = 0.027) in China. However, another study by Schrijvers et al. [9] demonstrated no significant relationship between HIF-1*α* expression and an early clinical stage in SCC of the glottis. However, the difference in clinical stage between their study, which focused on early stage patients, and our study, which included predominantly advanced stage patients, makes it difficult to compare our results.

In other malignancies, Cao et al. [10] have demonstrated a significant correlation between HIF-1*α* expression and an advanced clinical stage in colorectal carcinoma. Lin et al. [11] have also reported a statistically significant correlation between HIF-1*α* expression and tumor size, regional metastasis, and an advanced clinical stage in oral cavity SCC. Another study by No et al. [12] examined the relationship between HIF-1*α* and vascular endothelial growth factor (VEGF) expression in cervical carcinomas. They found no statistically significant relationship between HIF-1*α* expression and tumor size or regional metastasis, but they concluded that cervical carcinoma carcinogenesis is influenced by HIF-1*α*, although the exact mechanism remained unclear.

In this study, we found no associations between HIF-1*α* expression and LSCC stage or differentiation, indicating that other biomarkers may be important in defining LSCC carcinogenesis. HIF-1*α* is induced by hypoxia in solid tumors and enables tumor cell adaptation to low oxygen conditions. However, HIF-1*α* expression is regulated by tightly controlled expression and degradation cycles. Toffoli and Michiels [13] have shown that there are hypoxic and nonhypoxic regions contained within individual tumors. Furthermore, hypoxic areas can undergo a process of reoxygenation to restore normoxic conditions; HIF-1*α* that is stable in hypoxic conditions would be degraded during the process of reoxygenation. As such, tumor areas that have undergone reoxygenation to restore oxygen conditions would stain negative for HIF-1*α* expression.

Complex mechanisms of carcinogenesis underlie the progression of LSCC from an early clinical stage to an advanced clinical stage. During this transition, complex changes occur in the tumors, and many studies are being conducted to improve our understanding of these processes. When solid tumors encounter hypoxic conditions and HIF-1*α* is expressed, a number of processes occur to achieve tumor progression and an improved clinical stage. The expression of HIF-1*α* in hypoxic conditions triggers genomic instability, angiogenesis, apoptosis, immune evasion, tumor invasion and metastasis, and the activation of glycolysis. All of these processes produce changes in the cellular and molecular environment and are underpinned by further complex mechanisms [13, 14].

According to Poon et al. [14], the activation of HIF-1*α* requires, in addition to hypoxic conditions, changes in tumor microenvironment stimuli from a number of factors, including growth factors, the von-Hippel-Lindau tumor suppressor protein, succinate dehydrogenase, p53, and other proteins. HIF-1*α* protein expression in tumor cells triggers malignant progression through various processes, including angiogenesis, which is achieved through VEGF; alterations in glucose metabolism, which is achieved through glucose transporter 1; tumor cell survival, which is achieved though insulin-like growth factor-1; and metastasis, which is achieved through lysyl oxidase and plasminogen activator inhibitor-1. This study only measured the expression of HIF-1*α* protein, and many studies have failed to show a relationship between HIF-1*α* expression and clinical stage. However, given the complexity of the mechanisms that underlie the progression and development of LSCC, we anticipate that other factors will be shown to be correlated with LSCC clinical stage.

A study by No et al. [12] has shown no statistically significant relationship between HIF-1*α* protein expression and LSCC tumor size or metastasis. It is assumed that the progression of a malignancy, which is usually characterized by an increased clinical stage, occurs through a number of complex mechanisms, not the action of one or two factors alone. Ruan et al. [15] have proposed that oxygen diffusion is sufficient for oxygen to travel to 100–180 *μ*m between the distal end of a capillary and a cell. An increased tumor volume, indicated by a higher T stage, will decrease the efficacy of oxygen diffusion in maintaining normoxia; hypoxic conditions and HIF-1*α* stabilization will occur in tumor cells that are located far from the distal end of the capillary. Our study did not show any significant association between HIF-1*α* protein expression and LSCC metastasis. This adds strength to the argument that HIF-1*α* is not the only protein that plays a role in LSCC carcinogenesis; HIF-1*α* may play an active role in the progression of malignancy, but other proteins may be required to accelerate tumor development and aggressiveness.

The only statistically significant relationship demonstrated in this study was the relationship between HIF-1*α* expression and gender (*p* = 0.047). In contrast, Cao et al. [10] found no relationship between these variables, but their study cohort had a male : female patient ratio of approximately 1.5 : 1. In the present study, the male : female patient ratio was approximately 7 : 1. Since the gender distribution was not homogenous in our study, further studies with a more homogeneous distribution of patients are required to validate our finding.

## 5. Conclusions

Overall, our results emphasize the role of HIF-1*α* in carcinogenesis. Despite finding no statistically significant associations in the present study, HIF-1*α* and other biomarkers have been shown to contribute to the development and progression of LSCC. Further studies are required to clarify the molecular pathways underlying laryngeal cancer.

## Figures and Tables

**Table 1:** Patient characteristics.

Variable	Frequency (*n*)	Percentage (%)
Gender		
Male	41	87.2
Female	6	12.8
Age		
<60 years	24	51.1
≥60 years	23	48.9
Tumor location		
Glottis	42	89.4
Supraglottis	5	10.6
Clinical stage		
Early	4	8.5
Advanced	43	91.5
Differentiation		
Well	15	31.9
Moderate	24	51.1
Poor	8	17.0
HIF-1*α* expression		
Positive	29	61.7
Negative	18	38.3

**Table 2:** HIF-1*α* expression by clinical stage.

	*n*	HIF-1*α* expression		*p*
		Positive (*n* [%])	Negative (*n* [%])	
Tumor location				
Glottis	42	27 (64.3)	15 (35.7)	0.357
Supraglottis	5	2 (40.0)	3 (60.0)	
Clinical stage				
Early	4	2 (50.0)	2 (50.0)	0.631
Advanced	43	27 (62.8)	16 (37.2)	
Differentiation				
Well	15	7 (46.7)	8 (53.3)	0.158
Moderate	24	18 (75.0)	6 (25.0)	
Poor	8	4 (50.0)	4 (50.0)	

## Additional Points

*Summary Point*. HIF-1*α* can be demonstrated in LSCC.
